# Improving Iron Status Without Iron: Clinical Insights From Recombinant Human Lactoferrin Supplementation

**DOI:** 10.7759/cureus.107451

**Published:** 2026-04-21

**Authors:** Cassandra Evans, Ross Peterson, Jose Antonio, Anthony Clark

**Affiliations:** 1 College of Health Care Sciences, Nova Southeastern University, Davie, USA; 2 Scientific Affairs, Helaina Inc., New York, USA

**Keywords:** ferritin, iron alternative, iron-deficiency, iron parameters, lactoferrin

## Abstract

Helaina human lactoferrin (LF) (effera®) is a potential, well-tolerated alternative for improving iron status in women who present with chronic iron deficiency. This case series reports on two active women who had persistently low iron levels and poor response to, or intolerance of, standard orally administered iron supplements. The two individuals independently started oral ingestion of effera® at 100 mg/day for 6-20 weeks. Both showed notable increases in ferritin, with one also exhibiting marked increases in serum iron and transferrin saturation. The two participants reported better energy, fewer symptoms, and improved exercise capacity, all without gastrointestinal issues. These results align with growing evidence that LF supports iron homeostasis through receptor-mediated absorption, hepcidin-ferroportin modulation, and immune-balancing effects, potentially surpassing or working in synergy with conventional treatments. While limited to case reports, these observations support further controlled trials of effera® as an adjunct or alternative for iron deficiency, including non-anemic presentations.

## Introduction

Iron is an essential micronutrient needed for numerous biochemical pathways in the body. Iron is important for neurological development [1], reproductive health [2], and overall cellular function [3]. Iron’s chief functions in the body include oxygen transport, energy metabolism, and DNA synthesis [4,5]. Iron is required for the production of hemoglobin, an essential protein that transports oxygen throughout the body [5].

Iron is one of the most common micronutrient deficiencies worldwide and disproportionately affects women [6-8]. Iron needs and recommendations for women vary from 8 to 18 mg/day, depending on life stage [9]. Iron deficiency can present as iron deficiency anemia (IDA) or iron deficiency without anemia (IDWA) and may also coexist with anemia of inflammation. In IDA, low iron availability impairs hemoglobin production [10]. In anemia of inflammation, iron may be present but remains biologically unavailable due to elevated hepcidin, a regulatory hormone that reduces intestinal iron absorption and limits iron release from tissues [11]. In IDWA, iron stores are depleted despite hemoglobin concentrations remaining within normal ranges [7].

Importantly, the absence of anemia does not rule out clinically meaningful iron deficiency. Women with IDWA may still experience fatigue, reduced concentration, impaired physical performance, and lower quality of life, and IDWA is estimated to affect approximately 15%-35% of women [7,8]. Even when standard iron indices appear acceptable, some individuals may remain functionally iron-deficient due to impaired iron absorption or utilization [11]. Early recognition and management of iron deficiency, including non-anemic presentations, is important in correcting chronic disruptions in iron balance [10,12].

Iron supplementation remains the standard treatment for iron deficiency; however, it is associated with many side effects, including gastrointestinal symptoms such as constipation, nausea, and abdominal discomfort [13,14]. Oral iron absorption is also relatively limited, and a substantial proportion of supplemental iron may remain unabsorbed [15]. In addition, iron supplementation may also acutely increase hepcidin, a key regulator of iron homeostasis, which can further reduce iron absorption from subsequent doses [15]. It is not uncommon for individuals to report no improvement despite prolonged use of iron supplements. As a result, alternative approaches that support iron metabolism have gained increasing interest.

Lactoferrin (LF) is an iron-binding glycoprotein naturally present in mammalian milk and various bodily secretions throughout the lifecycle [16]. It is particularly abundant in colostrum and is part of the whey protein fraction of milk. Structurally, LF contains two lobes capable of binding iron and exists in different forms depending on its iron saturation. Apo-lactoferrin (apo-LF) is the iron-free form, while holo-lactoferrin (holo-LF) contains bound iron, with native LF typically consisting of a mixture of both forms. LF has a high affinity for ferric iron (Fe³⁺) and can bind two ferric iron atoms, contributing to its role in shifting the gut microbiome [17] and limiting iron-mediated oxidative stress [16,18]. Several clinical studies have reported improvements in iron biomarkers, including ferritin and hemoglobin, following LF supplementation in populations with iron deficiency [19-24]. These findings support that LF may influence iron metabolism through mechanisms beyond simple iron supplementation.

Helaina human LF (effera®) is produced at commercial scale using the yeast *Komagataella phaffii*, which exhibits structural equivalency to native human LF [25]. Following comprehensive safety [26-30] and structural [25,31] evaluations, effera® has met the Generally Recognized As Safe (GRAS) standard for its intended use as a food ingredient. Furthermore, it is available for use in dietary supplements.

The present report describes two individuals with a history of persistent low iron status who independently initiated effera® supplementation and subsequently demonstrated improvements in iron-related biomarkers. These cases provide real-world observations that contribute to the growing interest in LF as a supportive strategy for iron metabolism.

## Case presentation

Two individuals with a history of persistent low iron status despite prior iron supplementation were observed following the initiation of effera®-containing supplements. Participant characteristics are provided in Table 1. 

**Table 1:** Participant characteristics BMI: body mass index; GI: gastrointestinal; THC: tetrahydrocannabinol; N/A: not applicable

	Case 1	Case 2
Age	31	39
BMI	Not provided	21.4
Relevant Health History	Hashimoto, prev. anemia	Anemia since the early 20s
GI Issues	N/A	N/A
Physical Activity (weekly)	3 hours strength training, ~24 hours cardio	6 hours structured exercise (Hiking, Pilates)
effera^®^ Dose (mg/day)	100	100 (within Super Core)
Duration (weeks)	20 weeks	6 weeks
Co-supplementation	Magnesium, taurine, omega-3, carnitine, glutathione, creatine	Sleep gummy (THC, melatonin, and L-theanine), vitamin C, zinc, vitamin D, and vitamin K

Case 1

A 31-year-old physically active female with a history of Hashimoto’s thyroiditis and prior anemia reported a longstanding pattern of low iron status beginning in early adulthood. The participant reported oral iron supplementation and even receiving iron infusions to treat IDA. Despite these interventions, she indicated that improvements in iron status were inconsistent and difficult to maintain. Baseline laboratory testing is presented in Table 2.

**Table 2:** Iron laboratory measures and subjective report of case studies 1 and 2 GI: gastrointestinal

Laboratory Parameter and Subjective Report		Case 1				Case 2				Reference Range	
		Baseline		20 weeks	Δ	Baseline	6 weeks	Δ			
Ferritin (ng/mL)		11		57	↑46	22	34	↑12		16-154 ng/mL	
Serum Iron (µg/dL)		135		Not provided	-	41	143	↑102		60-140 mcg/dL	
Transferrin Saturation (%)		41		Not provided	-	11	43	↑32		25%-35%	
Total Iron Binding Capacity (mcg/dL)		328		Not provided	-	367	336	↓31		250-450 mcg/dL	
C-Reactive Protein (mg/L)		Not provided		Not provided	-	1.7	0.8	↓0.9		<1 mg/L	
Subjective Energy Change		Self-reported improvements				Self-reported improvements				-	
GI Tolerance		Well tolerated				Well tolerated					
Other Subjective Changes		Improved hair growth				Improvements in symptoms (headaches, nausea), improved hair growth					

The participant independently initiated supplementation with effera® at a dose of 100 mg per day and continued this regimen for approximately 20 weeks. Supplement timing was not standardized and was taken when convenient. She continued to use several additional dietary supplements, including magnesium, taurine, omega-3 fatty acids, carnitine, glutathione, and creatine. Laboratory testing was obtained independently by the participant and shared voluntarily; testing was not requested or directed as part of a structured clinical protocol. Follow-up laboratory results showed an increase in ferritin from 11 ng/mL to 57 ng/mL (+46 ng/mL) during the supplementation period (20 weeks). The participant also reported subjective improvements in overall energy levels and exercise tolerance.

Case 2

A 39-year-old female with no diagnosed chronic medical conditions or prescription medication use reported a longstanding history of low iron status beginning in early adulthood. She described a pattern of persistently low ferritin levels throughout adulthood, at times reported to be below 10 ng/mL. However, hemoglobin concentrations were often within normal clinical ranges, and formal treatment was rarely recommended. Despite otherwise good health and a balanced diet including animal-source foods, she reported recurrent dizziness and multiple fainting episodes during this period.

Following the birth of her first child six years prior to the present observation, the participant reported a change in symptom pattern characterized by persistent vertigo, nausea, and severe fatigue during the postpartum period. At that time, ferritin was documented at 9 ng/mL, and she was advised to begin oral iron supplementation (Floradix® liquid iron), which she used intermittently thereafter when symptoms recurred. Over the following years, she reported cyclical worsening of symptoms following menstruation, including dizziness, nausea, and headaches. Although iron supplementation sometimes improved symptoms after several weeks, she reported difficulty maintaining consistent use due to gastrointestinal side effects, including constipation, bloating, and general intolerance to iron supplements.

Approximately six weeks prior to follow-up laboratory testing, the participant independently began using a dietary supplement containing effera® (Super Core; 100 mg effera® per day) at a dose of one scoop daily. During this period, she reduced the use of several other supplements due to nausea but continued use of vitamin C, zinc, vitamin D, vitamin K, and a nighttime sleep gummy containing melatonin, tetrahydrocannabinol (THC), and L-theanine. Laboratory testing was obtained independently by the participant and shared voluntarily; testing was not requested or directed as part of a structured clinical protocol. Baseline laboratory values are presented in Table 2. Follow-up laboratory results obtained approximately six weeks after initiating supplementation showed increases in ferritin (+12 ng/mL), serum iron (+102 µg/dL), and transferrin saturation (+32%) (Table 2). The participant also reported subjective improvements in overall energy and symptom burden during this period.

## Discussion

The present report describes two individuals with a history of persistent low iron status despite previous iron supplementation, who demonstrated improvements in iron-related biomarkers following effera®-containing supplementation. Both cases had a history of oral iron supplementation, which is widely used as the primary treatment for iron deficiency. Despite the prevalent use of iron supplementation, several factors limit its effectiveness. Gastrointestinal side effects frequently impair adherence, and the bioavailability of supplemental iron is relatively low [15,32]. Acute increases in hepcidin following iron ingestion may further reduce intestinal iron absorption, which may contribute to persistent low iron status in some individuals despite regular supplementation [11,15]. Both individuals showed improvements in iron-related biomarkers, notably ferritin, following supplementation with effera®. In Case 1, ferritin increased substantially over the course of supplementation, while Case 2 demonstrated concurrent increases in ferritin, serum iron, and transferrin saturation. C-reactive protein did not increase alongside ferritin in Case 2. This suggests that the rise in ferritin reflects improved iron status rather than an inflammatory response [33]. Both individuals reported subjective improvements in overall energy, mood, and hair growth during the supplementation period.

A common feature of both cases was the use of 100 mg per day of effera®. Produced through precision fermentation in *K. phaffii*, effera® is structurally identical to native human LF at the amino acid sequence level [25,29]. This feature allows it to mirror the biological functions of natural human LF [31], particularly its strong ability to bind and regulate iron [16]. Emerging clinical research further suggests that effera® may demonstrate advantages over bovine LF [25]. The authors of the present study suggest that the increases in iron biomarkers, specifically ferritin, observed in these two cases may be related to supplementation with effera®.

Clinically, LF supplementation enhances iron absorption and normalizes iron status in cases of deficiency by interacting with specific LF receptors on enterocytes [34]. LF influences key proteins involved in iron metabolism, including upregulation of ferroportin and transferrin receptors and downregulation of hepcidin [35]. LF may also support iron balance through healthy immune-balancing mechanisms, including reductions in interleukin-6 (IL-6), a cytokine known to suppress ferroportin and contribute to iron sequestration during inflammatory states [22]. LF has also been shown to modulate the hepcidin-ferroportin axis, where its immune interactions decrease hepcidin levels, improving iron utilization [22].

Clinical studies have reported improvements in hemoglobin, ferritin, and transferrin saturation following LF supplementation despite the relatively small amount of iron delivered by LF compared with conventional iron therapy [22,36]. Unlike ferrous iron, which can act as a prooxidant and contribute to oxidative reactions in the gastrointestinal tract, iron bound to LF exists primarily in the ferric form and may reduce the potential for iron-mediated oxidative damage [37]. In a randomized trial, Paesano et al. (2010) [38] reported significant increases in hemoglobin, serum iron, and ferritin following bovine LF supplementation in women with iron deficiency. Additionally, reductions in the pro-inflammatory cytokine IL-6 were reported. It is important to note that this study observed these improvements with LF supplementation that contained no additional iron. These findings are consistent with the present cases, in which improvements in iron biomarkers, specifically ferritin, were observed following LF supplementation.

This case series is not without limitations, including its observational nature and incomplete laboratory data in Case 1. The subjective improvements reported should be interpreted cautiously, as a placebo effect cannot be ruled out in the absence of a control group. Taken together, the magnitude of change, particularly in Case 1, should not be viewed as clinically definitive but rather as a preliminary signal that warrants further investigation.

## Conclusions

In summary, this case series suggest that daily supplementation with effera® at 100 mg may help improve iron-related biomarkers in women with chronically low iron levels who are unresponsive to standard iron therapy. Both participants experienced notable increases in ferritin and, in one instance, elevated serum iron and transferrin saturation. Additionally, they experienced subjective benefits, including greater energy, fewer symptoms, and improved exercise tolerance, without gastrointestinal discomfort or adverse effects. These observations align with clinical evidence demonstrating that LF supplementation supports iron homeostasis, often with better tolerability than conventional supplementation. Interpretation of these findings should be made cautiously, given the observational nature of the case reports. Although these individual reports are intriguing, larger, well-controlled clinical trials are needed to confirm effera®’s efficacy, optimal dose, and long-term safety across broader populations.

## References

[1] Armitage AE, Moretti D (2019). The importance of iron status for young children in low- and middle-income countries: a narrative review. Pharmaceuticals (Basel).

[2] Tulenheimo-Silfvast A, Ruokolainen-Pursiainen L, Simberg N (2025). Association between iron deficiency and fertility. Acta Obstet Gynecol Scand.

[3] Perera DN, Palliyaguruge CL, Eapasinghe DD (2025). The role of iron as a micronutrient in key biological functions, health and diseases in human. Nutr Bull.

[4] Galy B, Conrad M, Muckenthaler M (2024). Mechanisms controlling cellular and systemic iron homeostasis. Nat Rev Mol Cell Biol.

[5] Cairo G, Bernuzzi F, Recalcati S (2006). A precious metal: iron, an essential nutrient for all cells. Genes Nutr.

[6] Auerbach M, DeLoughery TG, Tirnauer JS (2025). Iron deficiency in adults: a review. JAMA.

[7] Özbilen M, Kaya Y (2025). Beyond anemia: a comprehensive analysis of iron deficiency symptoms in women and their correlation with biomarkers. BMC Womens Health.

[8] Sholzberg M, Hillis C, Crowther M, Selby R (2025). Diagnosis and management of iron deficiency in females. CMAJ.

[9] Friedman AJ, Chen Z, Ford P (2012). Iron deficiency anemia in women across the life span. J Womens Health (Larchmt).

[10] Takami T, Sakaida I (2011). Iron regulation by hepatocytes and free radicals. J Clin Biochem Nutr.

[11] Gafter-Gvili A, Schechter A, Rozen-Zvi B (2019). Iron deficiency anemia in chronic kidney disease. Acta Haematol.

[12] Moss AS, Pakbaz Z (2025). Iron deficiency-more than just anemia: a literature review. J Community Hosp Intern Med Perspect.

[13] Pantopoulos K (2024). Oral iron supplementation: new formulations, old questions. Haematologica.

[14] Tan X, Tian Y, Zhang T (2026). Oral iron preparations: gastrointestinal adverse events and medication adherence in female patients with iron deficiency anemia. Front Pharmacol.

[15] Moretti D, Goede JS, Zeder C (2015). Oral iron supplements increase hepcidin and decrease iron absorption from daily or twice-daily doses in iron-depleted young women. Blood.

[16] Kell DB, Heyden EL, Pretorius E (2020). The biology of lactoferrin, an iron-binding protein that can help defend against viruses and bacteria. Front Immunol.

[17] Peterson DR, van der Made J, Kaplan N (2026). Effects of human lactoferrin (effera®) at two doses versus bovine lactoferrin on the adult gut microbiome and fecal short-chain fatty acids: a randomized, double-blind trial (Preprint). Medrxiv.

[18] Kowalczyk P, Kaczyńska K, Kleczkowska P, Bukowska-Ośko I, Kramkowski K, Sulejczak D (2022). The lactoferrin phenomenon - a miracle molecule. Molecules.

[19] Paesano R, Pacifici E, Benedetti S, Berlutti F, Frioni A, Polimeni A, Valenti P (2014). Safety and efficacy of lactoferrin versus ferrous sulphate in curing iron deficiency and iron deficiency anaemia in hereditary thrombophilia pregnant women: an interventional study. Biometals.

[20] Paesano R, Pietropaoli M, Gessani S, Valenti P (2009). The influence of lactoferrin, orally administered, on systemic iron homeostasis in pregnant women suffering of iron deficiency and iron deficiency anaemia. Biochimie.

[21] Paesano R, Torcia F, Berlutti F, Pacifici E, Ebano V, Moscarini M, Valenti P (2006). Oral administration of lactoferrin increases hemoglobin and total serum iron in pregnant women. Biochem Cell Biol.

[22] Lepanto MS, Rosa L, Cutone A, Conte MP, Paesano R, Valenti P (2018). Efficacy of lactoferrin oral administration in the treatment of anemia and anemia of inflammation in pregnant and non-pregnant women: an interventional study. Front Immunol.

[23] Cutone A, Rosa L, Lepanto MS (2017). Lactoferrin efficiently counteracts the inflammation-induced changes of the iron homeostasis system in macrophages. Front Immunol.

[24] Rosa L, Lepanto MS, Cutone A (2020). Influence of oral administration mode on the efficacy of commercial bovine lactoferrin against iron and inflammatory homeostasis disorders. Biometals.

[25] Lu X, Cummings C, Osuala UA (2024). Characterization of recombinant human lactoferrin expressed in Komagataella phaffii. Analyst.

[26] Vishwanath-Deutsch R, Dallas DC, Besada-Lombana P (2024). A review of the safety evidence on recombinant human lactoferrin for use as a food ingredient. Food Chem Toxicol.

[27] Peterson RD, Guarneiri LL, Adams CG (2024). A randomized, double-blind, controlled trial to assess the effects of lactoferrin at two doses vs. active control on immunological and safety parameters in healthy adults (Preprint). medRxiv.

[28] Peterson R, Crawford RB, Blevins LK (2024). Dose range-finding toxicity study in rats with recombinant human lactoferrin produced in Komagataella phaffii. Int J Toxicol.

[29] Anaya Y, Rosario Martinez R, Goodman RE (2024). Evaluation of the potential food allergy risks of human lactoferrin expressed in Komagataella phaffii. Front Immunol.

[30] Peterson RD, Guarneiri LL, Adams CG (2025). A randomized, double-blind, controlled trial to assess the effects of lactoferrin at two doses vs. active control on immunological and safety parameters in healthy adults. Int J Toxicol.

[31] Kim BJ, Kuhfeld RF, Haas JL (2024). Digestive profiles of human milk, recombinant human and bovine lactoferrin: comparing the retained intact protein and peptide release. Nutrients.

[32] Bloor SR, Schutte R, Hobson AR (2021). Oral iron supplementation - gastrointestinal side effects and the impact on the gut microbiota. Microbiol Res.

[33] World Health Organization (2020). WHO Guideline on Use of Ferritin Concentrations to Assess Iron Status in Individuals and Populations [Internet]. https://www.ncbi.nlm.nih.gov/books/NBK569877/.

[34] Conesa C, Bellés A, Grasa L, Sánchez L (2023). The role of lactoferrin in intestinal health. Pharmaceutics.

[35] Ianiro G, Rosa L, Bonaccorsi di Patti MC, Valenti P, Musci G, Cutone A (2023). Lactoferrin: from the structure to the functional orchestration of iron homeostasis. Biometals.

[36] Christofi MD, Giannakou K, Mpouzika M, Merkouris A, Stylianide MV, Charalambous A (2024). The effectiveness of oral bovine lactoferrin compared to iron supplementation in patients with a low hemoglobin profile: a systematic review and meta-analysis of randomized clinical trials. BMC Nutr.

[37] Lönnerdal B, Bryant A (2006). Absorption of iron from recombinant human lactoferrin in young US women. Am J Clin Nutr.

[38] Paesano R, Berlutti F, Pietropaoli M, Goolsbee W, Pacifici E, Valenti P (2010). Lactoferrin efficacy versus ferrous sulfate in curing iron disorders in pregnant and non-pregnant women. Int J Immunopathol Pharmacol.

